# Frailty and Age as Predictors of Mortality in Acute Myocardial Infarction Complicated by Cardiogenic Shock

**DOI:** 10.1016/j.jacadv.2025.102118

**Published:** 2025-09-17

**Authors:** M. Wasim Ahmadzai, Joakim Bo Kunkel, Peter Laursen Graversen, Anika Klein, Karoline Korsholm Jeppesen, Emma Illum, Jakob Josiassen, Ole Kristian Lerche Helgestad, Henrik Schmidt, Lene Holmvang, Lisette Okkels Jensen, Emil Fosbøl, Hanne Berg Ravn, Jacob Eifer Møller, Christian Hassager

**Affiliations:** a Department of Cardiology, Copenhagen University Hospital, Rigshospitalet, Copenhagen, Denmark; b Department of Cardiology, Odense University Hospital, Odense, Denmark; c Department of Cardiothoracic Anaesthesia, Odense University Hospital, Odense, Denmark

**Keywords:** AMI, AMICS, cardiogenic shock, frailty, Hospital Frailty Risk Score, mortality

## Abstract

**Background:**

Cardiogenic shock is a serious complication of acute myocardial infarction (AMICS) with an in-hospital mortality rate of approximately 50%. Frailty is associated with poor outcomes in general but has been poorly investigated in AMICS.

**Objectives:**

The purpose of this study was to evaluate the independent and combined effects of frailty and age on mortality in AMICS patients.

**Methods:**

We conducted a retrospective observational study using the RETROSHOCK cohort, including 1,716 AMICS patients admitted to 2 tertiary university hospitals in Denmark (2010-2017). Frailty was assessed using the Hospital Frailty Risk Score, with frailty defined as a score ≥5. Patients were further stratified by age ≥70 and <70 years old. In a separate analysis, age was categorized into 5 groups at 10-year intervals. Mortality was assessed at 30 days, 1 year (30-day landmark), and 10 years (30-day landmark).

**Results:**

Frailty was present in 22% (n = 381). The median age was 69.5 years (IQR: 61-77). The 30-day mortality rate was 65% in frail patients compared to 51% in nonfrail patients, *P* < 0.001. The highest mortality was observed in patients aged ≥80 years (82%; *P* < 0.001) at 30 days, with more than 9-fold adjusted increased risk of death compared to nonfrail patients under 50 years (adjusted HR: 9.18; 95% CI: 4.66-18.1; *P* < 0.001). Combined analysis of age and frailty demonstrated 73% mortality in frail patients ≥70 years at 30 days and 82% at 10 years (30-day landmark). Frailty remained independently associated with increased risk of mortality (aHR: 1.84; 95% CI: 1.37-2.46; *P* < 0.001).

**Conclusions:**

Frailty and age are strong, independent predictors of mortality in AMICS.

Cardiogenic shock (CS) is a serious condition. It complicates approximately 10% of acute myocardial infarctions (AMI), especially those with extensive myocardial injury and has an in-hospital mortality of around 50%.^1^^,^^2^ Managing AMI complicated by CS (AMICS) requires extensive resources and involves multiple interventions ranging from immediate revascularization, continuous intravenous infusions, and dialysis to mechanical ventilation and mechanical circulatory support (MCS). While these treatments can be lifesaving, they may also cause harm or prove futile, requiring physicians to be careful about their potential impact on long-term quality of life. The prospect of undergoing physically demanding and sometimes complication-prone therapies makes prognostication a critical aspect of clinical decision-making. Despite numerous pharmacological and invasive treatment choices,^3^ studies validating the safety and effectiveness of these methods often excluded older frail patients.^4^^,^^5^ Moreover, recent data suggest that while MCS is beneficial in selected patients, it may be less beneficial in elderly patients.^6^ This complicates the application of evidence-based medicine in a large, high-risk group.^4^^,^^7^ Frailty, a concept reflecting increased physiological vulnerability, may affect how patients respond to treatment during CS.^8^ Frailty is commonly associated with aging, presenting as heightened risk for falls, disability, hospitalization, and mortality. When these factors interact, the risk of adverse outcomes and mortality increases even further.^9^, ^10^, ^11^ Several tools are available to measure frailty.^10^, ^11^, ^12^ The Hospital Frailty Risk Score (HFRS) based on the International Classification of Diseases (ICD)-10th Revision (ICD-10) offers hospitals and health systems an economical and systematic approach to screen for frailty.^12^ Unlike other frailty assessment tools, which often require direct patient evaluation, the HFRS can be calculated using routinely collected hospital data, making it particularly useful in critically ill patients who cannot be assessed in their habitual state. A recent study found that frailty is common in AMICS and linked to worse in-hospital outcomes, including higher mortality and lower use of revascularization and mechanical support.^13^ Our study builds on these findings by analyzing a clinically validated European consecutive AMICS cohort, coupling it with nationwide health registry data to allow for a detailed assessment of frailty and its impact on AMICS outcomes. We hypothesize that frailty, as assessed by the HFRS, in addition to age, is independently associated with increased mortality.

## Methods

### Study population

This study is based on data from a retrospective, multicenter observational cohort (RETROSHOCK) that has previously been described in detail.^2^^,^^14^ In brief, the database covers all patients with AMICS who were admitted to one of 2 tertiary University centers in Denmark (Odense University Hospital and Copenhagen University Hospital Rigshospitalet) from 2010 to 2017, with an uptake covering two-thirds of all Danish patients (3.9 million citizens). The screening algorithm has also been described in detail.^2^ A contemporary definition of myocardial infarction was used,^15^^,^^16^ and the diagnosis of CS was validated through individual chart review, where the following criteria had to be met: 1) age ≥18 years; 2) persistent hypotension with systolic blood pressure ≤90 mm Hg for >30 minutes and/or use of vasoactive drugs and/or use of MCS; 3) signs of impaired organ perfusion (at least one of the following: altered mental status excluding medically induced sedation; cold/clammy skin; oliguria; arterial lactate ≥2.5 mmol/L); and 4) documented reduction in left and/or right ventricular function in the absence of hypovolemia, sepsis, anaphylaxis, pulmonary embolism, or primary valve dysfunction. Demographic, prehospital, clinical, and treatment characteristics were recorded for each patient in an electronic case report form.

### Definition of frailty and age

Patients were assigned a frailty status based on the HFRS, a numerical score derived by weighting selected ICD-10 diagnostic codes.^12^ Each ICD-10 code assigns a specific number of points, with a cutoff of 5 points indicating frailty. Patients with an HFRS ≥5 cumulated over 10 years before admission were classified as frail. This definition matches the original development and validation of the score.^12^ Patients aged 70 years or above were defined as “old,” otherwise “young.” The choice of 70 years as a threshold for defining older patients aligns with a recommendation from the European Society of Cardiology’s consensus document on frailty in cardiology, which highlights the relationship between frailty, advanced age, and cardiovascular risk.^17^ Thus, the population was divided into 4 groups: young + nonfrail, young + frail, old + nonfrail, and old + frail (Supplemental Figure 1). Patients were also categorized into 5 age groups at 10-year intervals to explore age-related trends.

### Registry-based follow-up and outcomes

Every Danish citizen is identified through a unique Civil Personal Registration number—all patients in the database can therefore be coupled with the National Patient Register, enabling further characterization regarding prior hospital contacts and associated diagnoses (ICD-10 and ICD-8th Revision). The outcome was all-cause mortality at 30 days, 1 year (landmarked at 30 days), and 10 years (landmarked at 30 days), determined through the Danish National Cause of Death Register. No individuals were censored due to loss to follow-up; all were followed until death or the administrative censoring date of December 31, 2024.

### Statistical analysis

Differences in patient characteristics by group were calculated using the chi-squared test or Fisher exact test for categorical variables, and the Kruskal-Wallis rank sum test for continuous variables as appropriate. Categorical variables are presented as n (%), whereas continuous variables are presented as median (IQR) or mean ±SD with 95% CI as appropriate. Kaplan-Meier analysis was used to determine crude mortality rates in each group, and the log-rank test was used to assess for significant differences between groups. Benjamini-Hochberg correction was applied when testing pairwise mortality differences. Univariable and multivariable Cox proportional hazards regression analyses were used to estimate unadjusted and adjusted mortality risks in each group expressed as HRs and 95% CIs. Clinically relevant covariates were selected for adjusted analysis (sex, diabetes, smoking, dyslipidemia, history of ischemic heart disease, left ventricular ejection fraction, arterial lactate level on admission, out-of-hospital cardiac arrest (OHCA), and attempted revascularization). Multivariable logistic regression was used to identify predictors of revascularization. To evaluate the relationship between frailty and mortality, we used penalized splines in a Cox proportional hazards regression model to explore potential nonlinear effects. Age groups (<50, 50-59, 60-69, 70-79, and ≥80 years) were treated as a stratification variable. Predicted HRs with 95% CIs were calculated for HFRS values ranging from 0 to 25 within each age group and visualized as smooth curves stratified by age. All statistical analyses were performed with *R* (v4.4.3, R Core Team 2024) with the *survival* package (v3.7-0) for primary analyses.

### Ethical approval

The database and study were approved by the Danish Patient Safety Authority (previously overseen by the Danish Health and Medicines Authority; case no. 3-3013-1133/1) and registered with the Danish Data Protection Agency (file nos. 16/7381 and 18/23756).

## Results

Baseline characteristics of patients are summarized in Table 1. A total of 1,716 patients were included, with 1,710 available for registry-based follow-up. Among these, 381 patients (22%) were characterized as frail (HFRS ≥5), while 1,329 (78%) were nonfrail. A total of 923 (54%) were classified as young (<70 years), and 787 (46%) were classified as old (≥70 years). Median follow-up among survivors was 9.3 years (IQR: 7.7-11.6) (Central Illustration).

**Table 1:** Baseline Characteristics of Patients in RETROSHOCK, Stratified According to Frailty (Hospital Frailty Risk Score ≥5) and Old Age (≥70 Years)

	N	Age <70 Years		*P* Value	Age ≥70 Years		*P* Value
		Nonfrail (n = 777)	Frail (n = 146)		Nonfrail (n = 552)	Frail (n = 235)	
Male, n (%)	1,710	644 (83%)	114 (78%)	ns	380 (69%)	135 (57%)	0.005
Age, y, median (Q1, Q3)	1,710	60 (53, 65)	62 (56, 66)	0.027	77 (73, 81)	77 (73, 83)	ns
Age group, n (%)	1,710			ns			ns
<50		116 (15%)	18 (12%)		-	-	
50-59		242 (31%)	35 (24%)		-	-	
60-69		419 (54%)	93 (64%)		-	-	
70-79		-	-		372 (67%)	143 (61%)	
80+		-	-		180 (33%)	92 (39%)	
Active smoking, n (%)	1,674	257 (34%)	50 (35%)	ns	123 (23%)	33 (14%)	0.021
Body mass index, kg/m^2^, median (Q1, Q3)	1,184	26.3 (24.2, 29.4)	25.2 (23.1, 28.1)	0.027	25.1 (23.1, 27.8)	24.7 (22.7, 27.6)	ns
Hypertension, n (%)	1,615	293 (40%)	78 (55%)	0.001	319 (61%)	150 (70%)	0.044
Hyperlipidemia, n (%)	1,598	205 (28%)	69 (49%)	<0.001	180 (35%)	103 (49%)	0.001
Diabetes mellitus (type 1 and 2), n (%)	1,620	122 (16%)	43 (30%)	<0.001	98 (19%)	55 (26%)	ns
History of acute myocardial infarction, n (%)	1,040	79 (11%)	40 (28%)	<0.001	80 (15%)	53 (24%)	0.009
History of ischemic heart disease, n (%)	1,642	166 (22%)	63 (44%)	<0.001	159 (30%)	93 (42%)	0.002
History of peripheral arterial disease	1,614	36 (4.9%)	26 (18%)	<0.001	47 (9.1%)	32 (15%)	0.050
Chronic lung disease, n (%)	1,614	41 (5.5%)	17 (12%)	0.018	82 (16%)	36 (17%)	ns
History of stroke, n (%)	1,618	17 (2.3%)	31 (22%)	<0.001	41 (7,4%)	46 (22%)	<0.001
Out-of-hospital cardiac arrest, n (%)	1710	434 (56%)	58 (40%)	<0.001	175 (32%)	53 (23%)	0.020
Systolic blood pressure, mm Hg median (Q1, Q3)	1,645	85 (74, 93)	86 (76, 95)	ns	83 (72, 90)	84 (72, 90)	ns
Diastolic blood pressure, mm Hg median (Q1, Q3)	1,558	54 (46, 60)	54 (48, 60)	ns	50 (43,60)	50 (44, 60)	ns
Heart rate, beats/min median (Q1, Q3)	1,477	84 (68, 100)	85 (72, 105)	ns	87 (71, 104)	82 (68, 100)	ns
Left ventricular ejection fraction, %	1,011	30 (20,40)	25 (15,35)	ns	30 (20,40)	25 (20, 35)	ns
Arterial lactate level, mmoL/L, median (Q1, Q3)	1,389	6.0 (3.2, 10.6)	5.7 (3.1,8.6)	ns	4.8 (2.9,8.4)	5.6 (3.2,7.8)	ns
Direct transfer to invasive center, n (%)	1,706	588 (76%)	84 (58%)	<0.001	339 (61%)	129 (55%)	ns
Revascularization attempt (PCI and/or CABG), n (%)	1,708	725 (93%)	122 (84%)	<0.001	454 (82%)	176 (75%)	0.038
Multivessel disease, n (%)	1,466	375 (53%)	81 (66%)	0.012	291 (65%)	118 (66%)	ns
TIMI flow 3 after PCI, n (%)	1,169	79 (14%)	15 (15%)	ns	78 (22%)	27 (19%)	ns
Use of intra-aortic balloon pump, n (%)	1,706	101 (13%)	15 (10%)	ns	59 (11%)	13 (5.6%)	0.043
Use of Impella, n (%)	1,707	128 (17%)	14 (9.6%)	ns	44 (8.0%)	20 (8.5%)	ns
Use of venoarterial extracorporeal membrane oxygenation, n (%)	1,705	46 (5.9%)	5 (3.4%)	ns	6 (1.1%)	-	ns

Patient characteristics stratified by age and frailty group.

CABG = coronary artery bypass graft; PCI = percutaneous coronary intervention; Q = quartile.

Counts below 3 are not reported due to data regulations and marked as -.

*P* values are pairwise, Bonferroni-corrected (x 2).

**Central Illustration:**
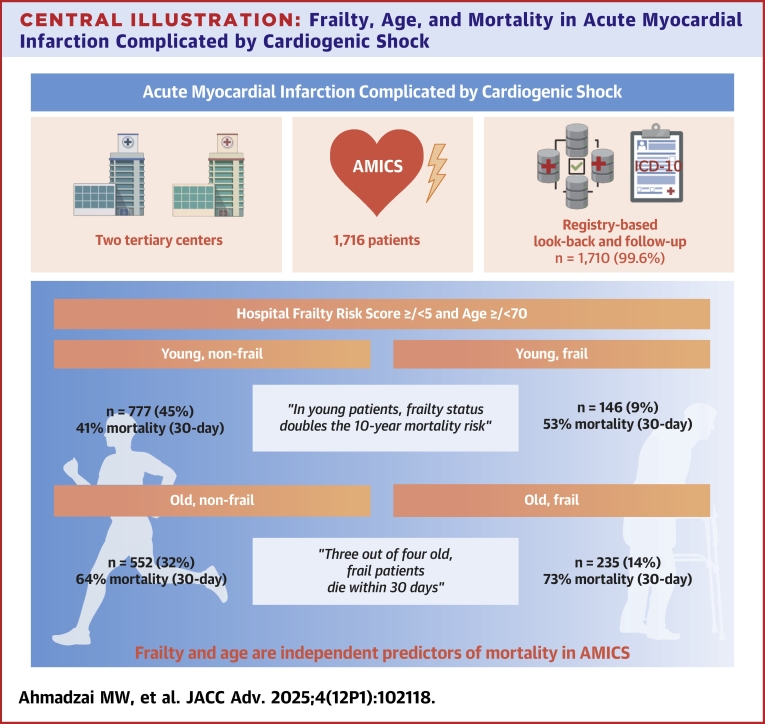
**Frailty, Age, and Mortality in Acute Myocardial Infarction Complicated by Cardiogenic Shock** This illustration summarizes the study design and main results. Patients with acute myocardial infarction complicated by cardiogenic shock were identified from 2 tertiary centers and followed through national registry linkages for up to 10 years. Frailty was assessed using the HFRS, and both frailty and age were independently associated with short- and long-term mortality. A graded relationship was observed between higher HFRS and mortality across age groups. AMI = acute myocardial infarction; OHCA = out-of-hospital cardiac arrest; other abbreviation as in Figure 1.

Among young patients, frailty was associated with a higher prevalence of hypertension (55% vs 40%; *P* = 0.001), hyperlipidemia (49% vs 28%; *P* < 0.001), diabetes mellitus (30% vs 16%; *P* < 0.001), and ischemic heart disease (44% vs 22%; *P* < 0.001). Similarly, frail old patients had more comorbidities than their nonfrail counterparts, including higher rates of hypertension (70% vs 61%; *P* = 0.044), hyperlipidemia (49% vs 35%; *P* = 0.001), and ischemic heart disease (42% vs 30%; *P* = 0.002).

Frailty was associated with a lower likelihood of direct transfer to an invasive center, with young frail patients being transferred less frequently than their nonfrail counterparts (58% vs 76%; *P* < 0.001). In multivariable logistic regression adjusting for clinical presentation and comorbidities, frailty was not independently associated with transfer patterns (adjusted OR [aOR] of direct transfer 0.85; 95% CI: 0.62-1.15; *P* = 0.30). Direct transfer was primarily predicted by OHCA presentation (aOR: 3.82; 95% CI: 2.87-5.12; *P* < 0.001). Revascularization attempts (percutaneous coronary intervention and/or coronary artery bypass grafting) were more common among nonfrail patients in both age groups, with the highest rates in young nonfrail patients (93%, *P* < 0.001) and the lowest in old frail patients (75%; *P* = 0.038).

Young patients had higher observed prevalences of OHCA compared to old patients (56% vs 32%; *P* < 0.001), but within each age group, frailty was associated with lower OHCA rates (young: 40% vs 56%; *P* < 0.001, old: 23% vs 32%; *P* = 0.020).

Hemodynamic parameters showed no significant differences between frail and nonfrail patients within each age group.

**Figure 1:**
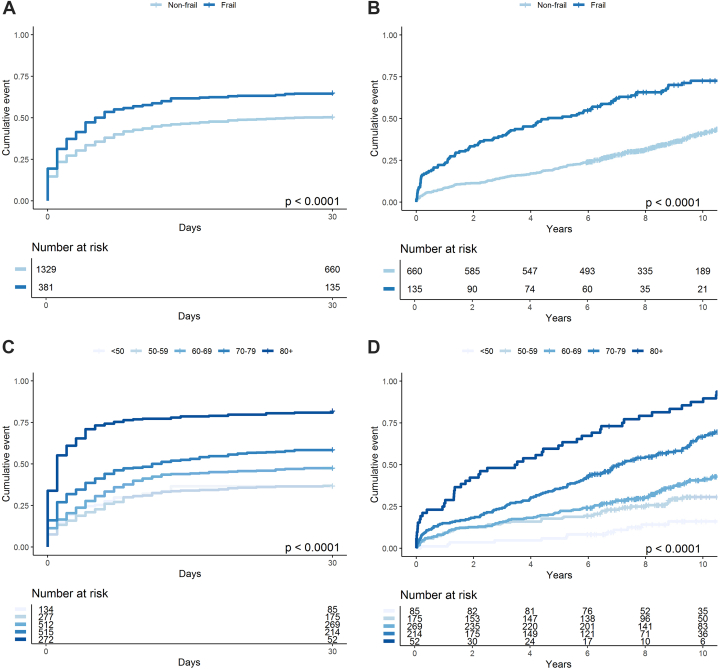
**Mortality by Frailty and Age in Acute Myocardial Infarction Complicated by Cardiogenic Shock** Short- and long-term mortality stratified by frailty and age groups. A and B show 30-day and 10-year mortality, respectively, by frailty status using the HFRS, with HFRS ≥5 defined as frail. C and D display 30-day and 10-year mortality by age group (<50, 50-59, 60-69, 70-79, and ≥80 years). Long-term mortality estimates use a 30-day landmark. All age groups and frailty strata were mutually exclusive. HFRS = Hospital Frailty Risk Score

**Figure 2:**
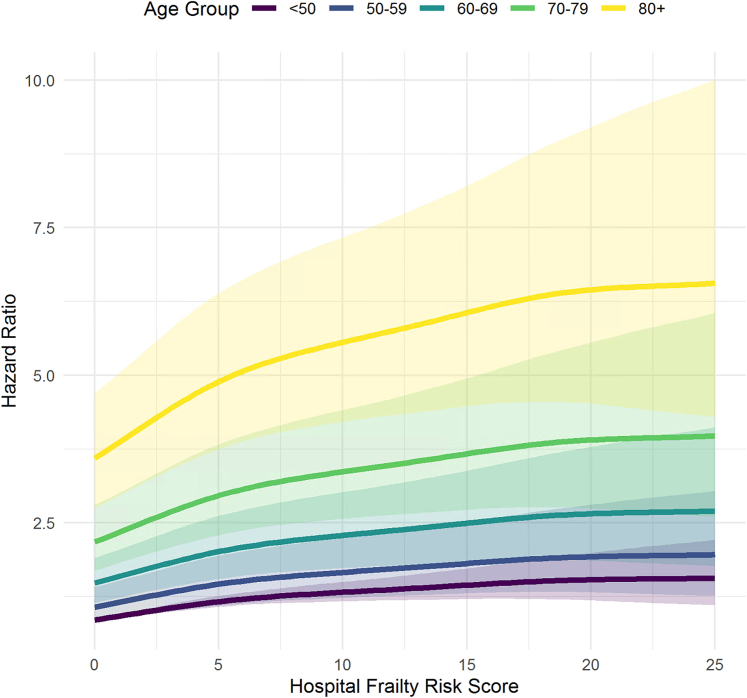
**Frailty-Associated Mortality Risk by Age Groups** Spline curves showing the association between the HFRS and the HR for all-cause mortality, stratified by age group (<50, 50-59, 60-69, 70-79, and ≥80 years). HRs were derived from Cox proportional hazards models using penalized spline regression with smoothing parameters determined by AIC optimization (effective df = 4). Each line represents one age stratum, with shaded areas representing 95% CIs. AIC = Akaike Information Criterion; other abbreviation as in Figure 1.

Mortality rates at 30 days, 1 year (30-day landmark), and 10 years (30-day landmark) are presented in Table 2, stratified by age, frailty status, and age groups. Overall, frail patients had significantly higher mortality than nonfrail patients at all time points (*P* < 0.001). The 30-day mortality rate was 65% in frail patients compared to 51% in nonfrail patients (Figure 1A). Within the frail cohort, higher HFRS scores were associated with progressively higher mortality (61.6%, 68.3%, and 73.1% for mild, moderate, and severe frailty, respectively), as illustrated in Figure 2). Among patients who survived the initial 30 days, mortality at 10 years (30-day landmark) reached 72% in frail patients vs 39% in nonfrail patients (*P* < 0.001) (Figure 1B).

**Table 2:** Mortality (30-Day and 1-Year [30-Day Landmark], and 10-Year [30-Day Landmark]) of Patients in RETROSHOCK Stratified According to Frailty (Hospital Frailty Risk Score ≥5), Old Age (≥70 Years) and Age Groups

	30-Day (95% CI)	*P* Value	30-Day Landmark			
			1 Year (95% CI)	*P* Value	10 Year (95% CI)	*P* Value
Age and frailty group						
Young, nonfrail	41% (37-44)	Ref	6.5% (4.2-8.8)	Ref	29% (24-33)	Ref
Young, frail	53% (44-60)	0.009	14% (5.8-22)	<0.001	62% (47-73)	<0.001
Old, nonfrail	64% (60-68)	<0.001	12% (7-16)	<0.001	64% (56-71)	<0.001
Old, frail	73% (66-78)	<0.001	30% (18-40)	<0.001	82% (68-90)	<0.001
Age group, y						
<50	37% (28-44)	Ref	1.2% (0-3.4)	Ref	16% (7.1-24)	Ref
50-59	37% (31-42)	<0.001	9.1% (4.8-13)	<0.001	30% (22-37)	<0.001
60-69	48% (43-52)	<0.001	8.6% (5.2-12)	<0.001	40% (34-47)	<0.001
70-79	59% (54-63)	<0.001	15% (9.7-19)	<0.001	64% (56-71)	<0.001
80+	82% (77-86)	<0.001	22% (9.8-33)	<0.001	85% (70-93)	<0.001
Frailty status						
Nonfrail	51% (48-53)	Ref	8.1% (6-10)	Ref	39% (35-44)	Ref
Frail	65% (60-70)	<0.001	22% (14-29)	<0.001	72% (62-79)	<0.001

*P* value = pairwise log-rank vs ref = Benjamini-Hochberg corrected.

Mortality increased with the age groups where patients aged ≥80 years had the highest 30-day mortality (82%, *P* < 0.001) (Figure 1C) and the highest 10-year mortality (85%, *P* < 0.001, 30-day landmark) (Figure 1D). In contrast, the youngest patients (<50 years) had the lowest mortality across all time points, with a 30-day mortality of 37% and a 10-year mortality of 16% (30-day landmark).

The combined analysis of age and frailty demonstrated that young and nonfrail patients had the lowest mortality, while old frail patients had the highest mortality at every follow-up point. At 30 days, mortality in old frail patients was 73% (*P* < 0.001) (Supplemental Figure 2A). Among those who survived the initial 30 days, mortality rose to 82% at 10 years (30-day landmark) (Table 2, Supplemental Figure 2B). Among patients who died within 30 days, 97% of frail patients and 96% of nonfrail patients died during the index hospitalization (*P* = 0.80).

Multivariable logistic regression analysis identified predictors of attempted revascularization. After adjustment for clinical characteristics, all groups maintained significantly lower odds of revascularization compared to young nonfrail patients: young frail (aOR: 0.35; 95% CI: 0.19-0.67; *P* = 0.001), old nonfrail (aOR: 0.34; 95% CI: 0.21-0.54; *P* < 0.001), and old frail (aOR: 0.25; 95% CI: 0.14-0.43; *P* < 0.001). When stratified by age, frailty was independently associated with lower odds of revascularization in patients <70 years (aOR: 0.37; 95% CI: 0.19-0.72; *P* = 0.003) but not in patients ≥70 years (aOR: 0.71; 95% CI: 0.44-1.16; *P* = 0.200).

Spline analysis demonstrated a significant nonlinear relationship between frailty on a continuous scale and mortality (*P* < 0.001 for all spline terms). Higher frailty scores were associated with increased mortality risk across all age groups (Figure 2). The effect of frailty was more pronounced in older patients (≥70 years), where incremental increases in frailty scores resulted in greater increases in mortality risk. In the overall cohort, a formal test for interaction between frailty and age did not reach statistical significance (*P* = 0.063), suggesting limited evidence of effect modification. However, in stratified analyses, frailty had a greater relative impact in older patients (≥70 years; HR: 1.26; *P* = 0.005), with a significant interaction term (*P* = 0.013). In contrast, in younger patients (<70 years), frailty was not significantly associated with mortality (HR: 1.14; *P* = 0.080), and the interaction term was nonsignificant (*P* = 0.20).

Cox proportional hazards regression was used to analyze long-term mortality risk over a follow-up period of up to 10 years (Table 3). In univariable analysis, both age and frailty were associated with increased mortality risk. When evaluated per decade, mortality risk progressively increased with age. In adjusted models accounting for sex, diabetes, smoking, dyslipidemia, history of ischemic heart disease, left ventricular ejection fraction, arterial lactate level, OHCA, and attempted revascularization during index admission, a significant association between age and increased mortality risk persisted. In the primary Cox model, one HFRS point corresponded to the mortality risk of 0.74 years of aging. In the 30-day landmark model, the effect increased to 1.24 years per point.

**Table 3:** Cox Proportional Hazards Regression Models for 10-Year Mortality Risk (30-Day Landmark), Stratified According to Frailty (Hospital Frailty Risk Score ≥5) and Old Age (≥70 Years)

10-Year Mortality Risk	Unadjusted			Adjusted			
	HR	95% CI	*P* Value	HR	95% CI	*P* Value	
Age and frailty group							
Young, nonfrail	Reference	-		Reference	-	-	a
Young, frail	2.88	2.03-4.10	<0.001	2.43	1.56-3.78	<0.001	
Old, nonfrail	2.97	2.32-3.81	<0.001	2.90	2.14-3.92	<0.001	
Old, frail	5.41	3.90-7.51	<0.001	4.59	3.07-6.85	<0.001	
Age group, y							
<50	Reference	-	-	Reference	-	-	b
50-59	2.31	1.23-4.34	0.009	1.91	0.99-3.69	0.053	
60-69	3.28	1.80-5.95	<0.001	2.57	1.38-4.79	0.003	
70-79	6.42	3.55-11.6	<0.001	4.47	2.56-8.78	<0.001	
80+	12.7	6.71-24.1	<0.001	9.18	4.66-18.1	<0.001	
Frailty status							
Nonfrail	Reference	-	-	Reference	-	-	c
Frail	2.59	2.04-3.30	<0.001	1.84	1.37-2.46	<0.001	

a Adjusted for sex, diabetes, smoking, dyslipidemia, history of ischemic heart disease, left ventricular ejection fraction, arterial lactate level on admission, out-of-hospital cardiac arrest, and attempted revascularization during index admission.

b Adjusted for a + frailty status.

c Adjusted for a + age.

Patients aged ≥80 years had the highest adjusted risk of mortality (adjusted HR [aHR]: 9.18; 95% CI: 4.66-18.1; *P* < 0.001) compared to those <50 years. Being both old and frail was associated with an almost five-fold increased mortality risk compared to young nonfrail patients (aHR: 4.59; 95% CI: 3.07-6.85; *P* < 0.001). Frailty, independent of age, remained a significant predictor of mortality (aHR: 1.84; 95% CI: 1.37-2.46; *P* < 0.001).

## Discussion

In this retrospective study based on data from the RETROSHOCK database, we investigated the impact of frailty and age on mortality in patients with AMICS admitted to 2 tertiary centers in Denmark. This cohort represents a selected population of AMICS patients who survived to hospital admission. Our findings demonstrate that both frailty and age are strong, independent predictors of short- and long-term mortality, and that their interaction influences treatment patterns and outcomes. These results extend recent registry-based observations by focusing on a clinically validated AMICS population, combining HFRS assessment with nationwide follow-up over 10 years.

The association between frailty and revascularization differed by age group. In younger patients, frailty was independently associated with lower revascularization rates after adjusting for comorbidities and clinical presentation. This suggests that in younger patients, frailty itself, beyond its associated comorbidities, influences clinical decision-making, perhaps because frailty in the young represents a more severe deviation from expected physiological status. In contrast, among older patients, frailty did not independently predict revascularization after multivariable adjustment, suggesting that chronological age may already limit invasive interventions regardless of frailty status. These patterns should be interpreted cautiously, as our tertiary center cohort likely excludes the frailest patients who were deemed unsuitable for transfer. Importantly, frailty remained predictive of mortality after adjusting for revascularization status, indicating that its prognostic impact extends beyond invasive treatment decisions.

In our study, frail patients were less frequently transferred directly to an invasive center, particularly among younger patients, suggesting that frailty may influence triage decisions. These findings indicate that patient selection for revascularization in AMICS is closely linked to frailty status, likely reflecting a combination of clinical judgment, procedural risk assessment, and expected treatment benefit. This aligns with recent research showing that frail patients with acute coronary syndrome are less likely to undergo revascularization, despite some evidence suggesting that invasive management may offer survival benefits even in those at higher frailty risk.^18^

Our study demonstrated that frailty in elderly patients with AMICS is significantly associated with increased mortality. Regardless of age, frail patients had an excess 30-day mortality, with early deaths almost exclusively occurring during the index hospitalization in both groups. Multiple factors, including the severity of coronary artery disease, burden of comorbidities, adverse effects of medications, and the severity of CS likely influence the elevated risk observed in this population.^19^^,^^20^ The lower rates of OHCA observed in older and frail patients likely reflect survival bias, as these patients are less likely to survive to hospital admission. Jentzer et al investigated the relationship between age, shock severity, and mortality in patients with CS.^19^ Using the Society for Cardiovascular Angiography and Intervention shock classification, they found that 30-day survival was 53.3% and progressively declined as both age and Society for Cardiovascular Angiography and Intervention shock stage increased. Our findings align with this, as we observed a stepwise increase in mortality with every 10-year rise in age at all time points. A recent secondary analysis of the DanGer Shock trial further highlights the impact of age in AMICS management. While younger patients with ST-segment elevation myocardial infarction–related CS benefited from microaxial flow pump therapy, this benefit seemed to diminish in older patients, particularly those ≥77 years, where survival benefit was not evident.^6^ These findings underscore the importance of considering age and frailty when evaluating advanced treatment strategies, as the risks and benefits are not uniform across age groups.

Hypertension, diabetes, and cardiovascular disease often co-occur and contribute to frailty, reducing physiological resilience. In our study, being both old and frail was associated with a more than four-fold increase in mortality. Frail younger patients also had a significantly increased risk, though the effect was less pronounced. Bai et al reported similar associations using the HFRS in critically ill AMI patients, though their cohort did not focus on AMICS and used shorter follow-up.^21^ The worse outcomes among younger frail patients in our study may reflect diagnostic coding bias, where frailty is only captured in patients with extreme disease burdens. In contrast, frailty may serve as a more accurate marker of physiological vulnerability in older individuals.

These findings highlight that frailty interacts with age in shaping treatment decisions and prognosis. In the future, digital frailty assessments could support earlier risk stratification and guide resource allocation. Particularly in high-risk settings such as AMICS, having frailty status available before or at the point of admission may facilitate more individualized, evidence-informed care.

While our findings support the prognostic value of frailty, interpreting these results requires careful consideration of how frailty is measured. The HFRS, developed by Gilbert et al, offers an efficient tool for identifying frailty using administrative data.^12^ It can be automatically calculated using ICD-10 codes, eliminating the need for manual scoring. However, it is not applicable in systems that do not use ICD-10 and may miss patients with few or no prior hospitalizations. Sy et al aimed to externally validate the HFRS in critically ill patients and concluded it could not predict in-hospital mortality in that setting, whereas Gilbert et al found the HFRS useful for estimating 30-day in-hospital mortality in a broader population.^22^^,^^23^ Differences in age and illness severity likely contributed to this discrepancy.

Importantly, the HFRS has not been formally validated in patients under 70 years of age. This may explain the weaker association between frailty and mortality observed in our younger subgroup. Younger adults may be undercoded for frailty-related conditions, and frailty in this group may reflect different underlying pathophysiology (eg, substance use or psychiatric comorbidity). This could lead to misclassification, with the HFRS capturing only the most severely frail younger patients while missing those with mild to moderate vulnerability.

In our study, 22% were classified as frail (HFRS ≥5), while 78% were not. Jamil et al reported a frailty rate of 70.8% using the same HFRS threshold.^3^ This substantial difference can be attributed to several factors. First, our cohort consisted exclusively of clinically validated AMICS patients who survived to hospital admission and were referred to tertiary cardiac centers, introducing selection bias toward fitter individuals. Second, Jamil et al used the National Inpatient Sample database, which captures diagnoses from the index hospitalization, potentially including acute complications that inflate frailty scores. In contrast, our 10-year lookback period captured pre-existing conditions but may have underreported frailty due to differences between Danish public health registries and U.S. hospital discharge coding practices. Third, the frailest patients may not have been transferred to our tertiary centers due to perceived futility, further reducing observed frailty prevalence.

### Study limitations

This study has several limitations. First, as a retrospective observational analysis, residual confounding and selection bias may persist despite statistical adjustment. Generalizability may be limited to patients treated at tertiary cardiac centers. Patients deemed too frail for transfer may not be represented in this cohort.

Second, frailty was assessed using the HFRS, which is derived from administrative ICD-10 codes and does not capture all dimensions of frailty, such as cognitive function, physical performance, or social vulnerability. The 10-year lookback period may have included outdated diagnoses, and frailty status at the time of admission may have changed, especially in older individuals with dynamic health trajectories. Third, to the authors’ knowledge, the HFRS has not been formally validated in patients under 70 years of age. Undercoding of frailty-related diagnoses in younger adults may have led to underdetection of moderate frailty, attenuating observed associations. Moreover, frailty in younger patients may reflect alternative pathophysiological mechanisms (eg, psychiatric or substance use disorders) not captured by the score.

Fourth, only all-cause mortality was available for this analysis. Other outcomes of clinical relevance, such as rehospitalization, functional recovery, symptom burden, and quality of life, were not directly assessed. Data on treatment preferences or limitations of care (eg, do-not-resuscitate orders or palliative care involvement) were also unavailable, which may have influenced treatment allocation independently of clinical characteristics.

Fifth, patients directly admitted to tertiary centers differed from those transferred from other hospitals. Although stratified analyses were performed, the potential influence of referral bias remains, particularly if frailty affected the likelihood of transfer.

## Conclusions

Frailty is common among elderly patients with AMICS, and both age and frailty are independently associated with higher short- and long-term mortality. This study provides insight into the interplay between frailty, age, and outcomes in AMICS and may inform future strategies for individualized risk stratification and care allocation.

Perspectives **COMPETENCY IN MEDICAL KNOWLEDGE:** Frailty, measured using the HFRS, is an independent predictor of mortality in patients with CS and provides prognostic information beyond chronological age.

**COMPETENCY IN PATIENT CARE:** Frailty and comorbidities influence treatment decisions in CS by reflecting an overall physiological reserve. Early characterization could support more individualized care, helping clinicians target intensive therapies to those most likely to benefit and avoid potentially nonbeneficial or even futile interventions.

**COMPETENCY IN SYSTEMS-BASED PRACTICE:** Integrating frailty screening into clinical workflows may support consistent, system-level decision-making in critical care. Use of automated tools based on existing data could improve risk stratification, resource use, and coordination of care across institutions.

**TRANSLATIONAL OUTLOOK:** The HFRS offers a digital and efficient method for assessing frailty by using patient health records. As clinical data systems evolve into platforms, integration with artificial intelligence may further enhance frailty-based decision support. Further studies are needed to determine whether such tools can improve treatment selection and long-term outcomes in critically ill patients.

## Funding support and author disclosures

Dr Hassager has received research grants from the 10.13039/501100003554Lundbeck Foundation, The 10.13039/100007405Danish Heart Foundation, and The 10.13039/501100009708Novo Nordisk Foundation. Dr Møller has received research grants from the 10.13039/501100009708Novo Nordic Foundation and the Institutional Research Grant Abiomed, advisory board Boston Scientific. Funding for the database was provided by the 10.13039/100007405Danish Heart Foundation, the Research Fund of 10.13039/501100004196Odense University Hospital and 10.13039/501100005111Rigshospitalet, and a grant from Abiomed. All other authors have reported that they have no relationships relevant to the contents of this paper to disclose.
