# Supplementary material for: Frailty and Age as Predictors of Mortality in Acute Myocardial Infarction Complicated by Cardiogenic Shock
Source: JACC Adv. 2025 Sep 17;4(12):102118. doi: 10.1016/j.jacadv.2025.102118 (PMC12805165; doi:10.1016/j.jacadv.2025.102118)

**Supplemental Table 1: Hospital Frailty Risk Score Definition**

| **Diagnosis Description** | **Diagnosis Code** | **Points Awarded** |
| --- | --- | --- |
| Dementia in Alzheimer disease | F00 | 7,1 |
| Alzheimer's disease | G30 | 4 |
| Vascular dementia | F01 | 2 |
| Unspecified dementia | F03 | 2,1 |
| Other degenerative diseases of nervous system, not elsewhere classified | G31 | 1,2 |
| Parkinson's disease | G20 | 1,8 |
| Epilepsy | G40 | 1,5 |
| Convulsions, not elsewhere classified | R56 | 2,6 |
| Speech disturbances, not elsewhere classified | R47 | 1 |
| Transient cerebral ischemic attacks and related syndromes | G45 | 1,2 |
| Hemiplegia | G81 | 4,4 |
| Sequelae of cerebrovascular disease | I69 | 3,7 |
| Other cerebrovascular diseases | I67 | 2,6 |
| Cerebral infarction | I63 | 0,8 |
| Abnormalities of gait and mobility | R26 | 2,6 |
| Somnolence, stupor and coma | R40 | 2,5 |
| Senility | R54 | 2,2 |
| Problems related to care-provider dependency | Z74 | 1,1 |
| Problems related to social environment | Z60 | 1,8 |
| Problems related to life-management difficulty | Z73 | 0,6 |
| Other symptoms and signs involving the nervous and musculoskeletal systems (Tendency to fall) | R29 | 3,6 |
| Unspecified fall | W19 | 3,2 |
| Other fall on same level | W18 | 2,1 |
| Fall on same level from slipping, tripping, and stumbling | W01 | 0,9 |
| Fall on and from stairs and steps | W10 | 0,9 |
| Fall involving bed | W06 | 1,1 |
| Fracture of shoulder and upper arm | S42 | 2,3 |
| Fracture of femur | S72 | 1,4 |
| Fracture of lumbar spine and pelvis | S32 | 1,4 |
| Fracture of rib(s), sternum and thoracic spine | S22 | 1,8 |
| Osteoporosis without pathological fracture | M81 | 1,4 |
| Osteoporosis with pathological fracture | M80 | 0,8 |
| Spinal stenosis | M48 | 0,5 |
| Scoliosis | M41 | 0,9 |
| Polyarthrosis | M15 | 0,4 |
| Other arthrosis | M19 | 1,5 |
| Other joint disorders, not elsewhere classified | M25 | 2,3 |
| Hypotension | I95 | 1,6 |
| Syncope and collapse | R55 | 1,8 |
| Abnormalities of heartbeat | R00 | 0,7 |
| Other septicemia | A41 | 1,6 |
| Respiratory failure, not elsewhere classified | J96 | 1,5 |
| Pneumonia, organism unspecified | J18 | 1,1 |
| Pneumonitis due to solids and liquids | J69 | 1 |
| Unspecified acute lower respiratory infection | J22 | 0,7 |
| Other functional intestinal disorders | K59 | 1,8 |
| Other diseases of digestive system | K92 | 0,8 |
| Diarrhea and gastroenteritis of presumed infectious origin | A09 | 1,1 |
| Other bacterial intestinal infections | A04 | 1,1 |
| Dysphagia | R13 | 0,8 |
| Vitamin D deficiency | E55 | 1 |
| Deficiency of other B group vitamins | E53 | 1,9 |
| Symptoms and signs concerning food and fluid intake | R63 | 0,9 |
| Other disorders of urinary system (UTI, incontinence) | N39 | 3,2 |
| Retention of urine | R33 | 1,3 |
| Unspecified urinary incontinence | R32 | 1,2 |
| Complications of genitourinary prosthetic devices, implants, and grafts | T83 | 2,4 |
| Chronic renal failure | N18 | 1,4 |
| Acute renal failure | N17 | 1,8 |
| Unspecified renal failure | N19 | 1,6 |
| Cellulitis | L03 | 2 |
| Decubitus ulcer | L89 | 1,7 |
| Other bacterial agents as the cause of diseases classified to other chapters (secondary code) | B96 | 2,9 |
| Other anemias | D64 | 0,4 |
| Fever of unknown origin | R50 | 0,1 |

**Supplemental Figure 1: Study Flowchart**

**Supplemental Figure 2: Short-term and long-term mortality stratified by combined frailty (Hospital Frailty Risk Score ≥5) and age (≥70) status.**

A: 30-day mortality B: 10-year mortality (30-day landmark)


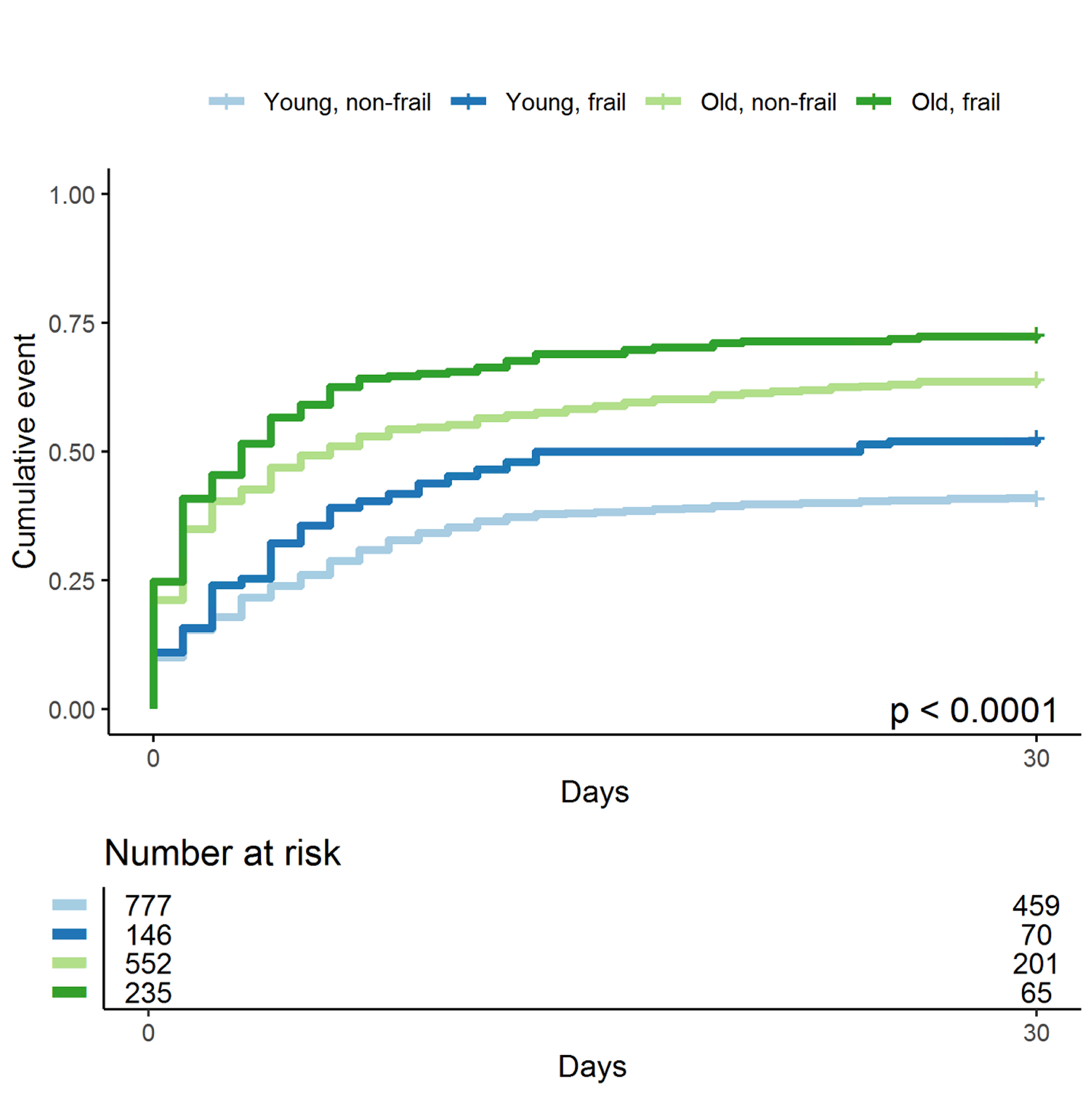

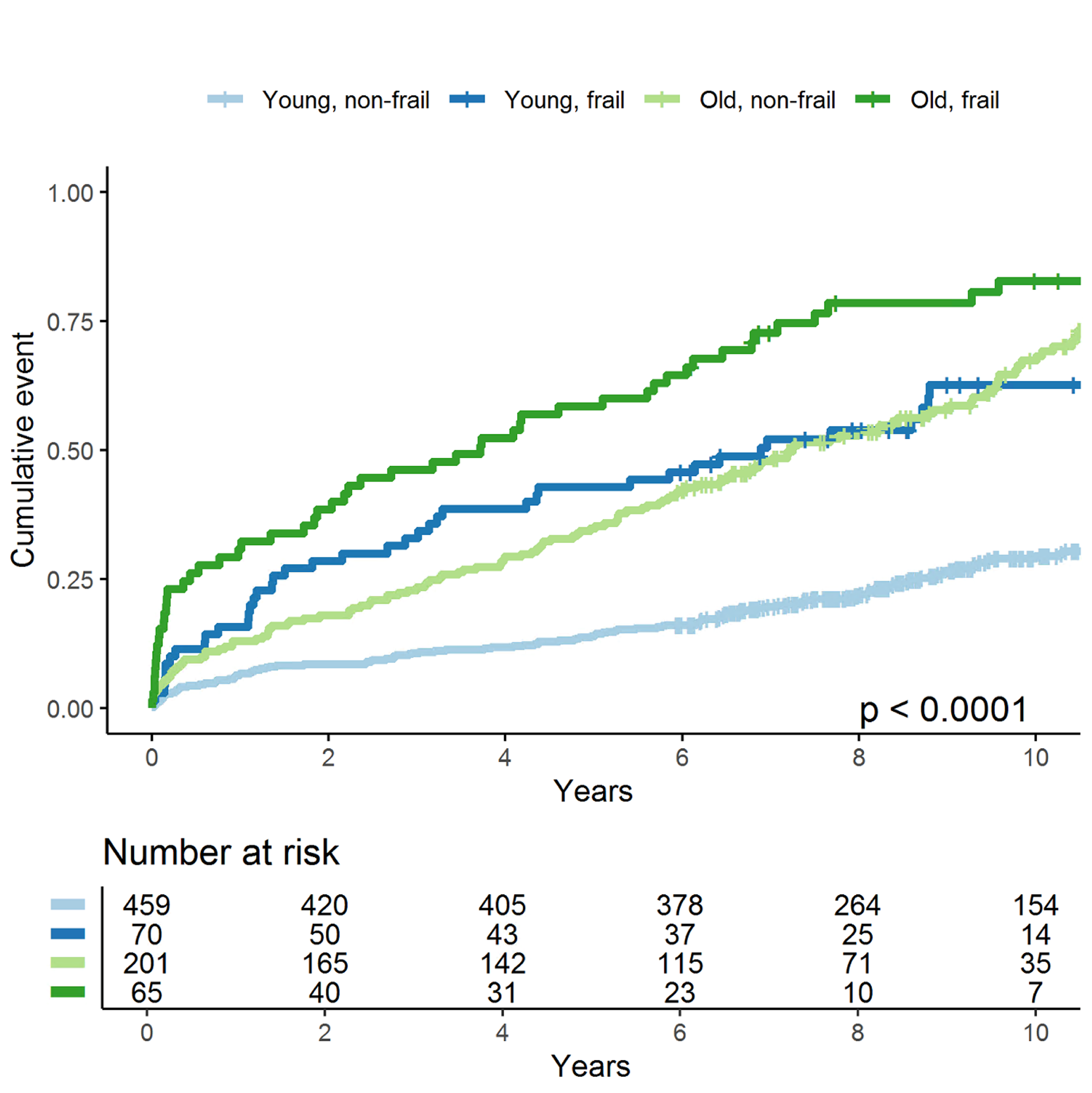

Supplement: Supplemental Material [file mmc1.docx]
